# Neurogenesis From Embryo to Adult – Lessons From Flies and Mice

**DOI:** 10.3389/fcell.2020.00533

**Published:** 2020-06-30

**Authors:** Helena Mira, Javier Morante

**Affiliations:** ^1^Instituto de Biomedicina de Valencia, Consejo Superior de Investigaciones Científicas, Valencia, Spain; ^2^Instituto de Neurociencias, Consejo Superior de Investigaciones Científicas y Universidad Miguel Hernandez, Alicante, Spain

**Keywords:** neurogenesis, neural stem cells, neural progenitors, niche, glia, intrinsic factors, extrinsic factors, adult neurogenesis

## Abstract

The human brain is composed of billions of cells, including neurons and glia, with an undetermined number of subtypes. During the embryonic and early postnatal stages, the vast majority of these cells are generated from neural progenitors and stem cells located in all regions of the neural tube. A smaller number of neurons will continue to be generated throughout our lives, in localized neurogenic zones, mainly confined at least in rodents to the subependymal zone of the lateral ventricles and the subgranular zone of the hippocampal dentate gyrus. During neurogenesis, a combination of extrinsic cues interacting with temporal and regional intrinsic programs are thought to be critical for increasing neuronal diversity, but their underlying mechanisms need further elucidation. In this review, we discuss the recent findings in *Drosophila* and mammals on the types of cell division and cell interactions used by neural progenitors and stem cells to sustain neurogenesis, and how they are influenced by glia.

## Neural Progenitors: Division Throughout Development and in Adult Neurogenic Niches

In all animals with a brain, from insects to humans, the complex functions the brain reliably carries out at every moment depend on its many neuronal and glial cell types being generated in the proper quantities and locations. Throughout the course of life, the production of new neurons that characterizes developmental stages also persists in two regions of the adult mammalian brain, the ventricular-subventricular or subependymal zone (SEZ) adjacent to the lateral ventricles and the subgranular zone (SGZ) of the hippocampal dentate gyrus. Recently there has been a great deal of controversy regarding the existence of adult neurogenesis in the human brain (Boldrini et al., 2018; Kempermann et al., 2018; Sorrells et al., 2018; Moreno-Jimenez et al., 2019), with confusion arising in part from technical problems and perhaps from interspecies differences in the dynamics of the process. Nevertheless, based on the accumulated evidence from previous work (Eriksson et al., 1998; Sanai et al., 2011; Spalding et al., 2013; Kempermann et al., 2018), it has become increasingly clear that adult humans probably generate new neurons only in the hippocampus and not in the SEZ.

For the purpose of this review, we will focus on the knowledge that deals with the neurogenic process in flies and mice. Here, we revisit recent findings on how neural stem cells (NSCs) divide to generate neuronal diversity during brain development and adulthood. We focus on the intrinsic and extrinsic mechanisms that explain the temporal and regional heterogeneity of neural progenitor and stem cells, and their progenies. We also summarize the role of niche glia in the early and late phases of neurogenesis and discuss their diversity. Whenever possible, we compare NSCs in *Drosophila* and rodents, at embryonic and larval stages and in adult neurogenic zones.

**FIGURE 1:**
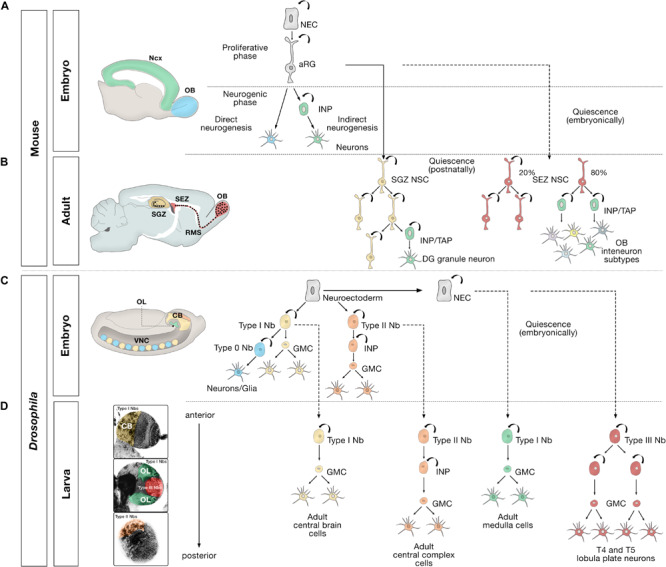
Embryonic, larval and adult neurogenesis in flies and mammals. **(A)** Overview of embryonic mammalian neurogenesis in the neocortex and olfactory bulb. In the developing dorsal pallium, the nervous system originates from neuroepithelial cells (NECs) that initially proliferate symmetrically before they transition to apical radial glial cells (aRG). aRGs give rise to neurons directly (blue), or indirectly (green) through intermediate neural progenitors (INP). Direct neurogenesis predominates in the olfactory bulb (OB, blue); indirect neurogenesis predominates in the neocortex (Ncx, green). **(B)** Adult mammalian neurogenesis in the subgranular zone (SGZ, yellow) and subependymal zone (SEZ, red). Quiescent postnatal neural stem cells in the SGZ (SGZ NSC) (yellow) undergo symmetric self-renewal before they give rise to transient amplifying cells, a type of intermediate neural progenitor (INP/TAP) (green) and differentiate into dentate gyrus granule neurons. Quiescent embryonic SEZ NSCs (red) are activated in the adult stage and undergo either symmetric self-renewing divisions (20%) or primarily produce INP/TAPs before differentiating into OB interneurons. **(C)** Different modes of division of neural progenitors in embryonic *Drosophila*. In the embryo, the nervous system originates from a neuroectoderm before they transit into neuroblasts (Nbs). Type 0 Nbs (blue) self-renew and produce a single ventral nerve cord (VNC) neuron at each division. Type I Nbs (yellow) self-renew and produce ganglion mother cells (GMC) that divide once to generate two cells in the central brain (CB, yellow) and VNC (yellow). Type II Nbs (orange) self-renew and produce intermediate neural progenitors (INP), which also self-renew multiple times before producing GMCs, which divide once and differentiate into central brain neurons (orange). Optic lobe cells (OL, green) originate from NECs. **(D)** Different modes of division of neural progenitors in the *Drosophila* larval brain. After the first, embryonic, wave of neurogenesis (shown in **C**), most of the remaining central brain and ventral nerve cord neuroblasts, and optic lobe NECs enter a quiescent state (dashed lines). In a second, larval, wave of neurogenesis, via ganglion mother cells (GMC), Type I Nbs in the central brain (CB, yellow region depicted in the larval brain) produce the majority of adult central brain cells, and Type II Nbs (orange region) produce the vast majority of central complex cells, an essential central brain region for sensorimotor integration (Pfeiffer and Homberg, 2014). Quiescent outer proliferation center (OPC) NECs are activated to transition into Type I Nbs (green region) and produce medulla cells in the OL. Type III Nbs (red) originate from NECs of the inner proliferation center (IPC), and undergo symmetric self-renewal to produce two identical progenies that retain the identity of neuroblasts and produce lobula plate cells in the OL.

The vast cell diversity in adult brains is mostly generated during the embryonic and larval stages in *Drosophila*, and in the embryonic and early postnatal stages in mammals, from a pool of neural progenitor and stem cells (Doe, 2017; Holguera and Desplan, 2018; Ramon-Canellas et al., 2018; Cardenas and Borrell, 2019; Morales and Mira, 2019; Obernier and Alvarez-Buylla, 2019). This pool initially includes neuroepithelial cells (NECs), which later produce multipotent NSCs (neuroblasts in *Drosophila*) and apical radial glia (aRG) (Figure 1). These cells span the apical–basal axis of the developing brain in mammals and have been best characterized in the neocortex (Gotz and Huttner, 2005; Kriegstein and Alvarez-Buylla, 2009; Brand and Livesey, 2011). By mid-gestation, a fraction of cortical, striatal, and septal radial glia diverge from other progenitors and are set aside as relatively quiescent cells that will give rise to postnatal and adult NSCs in the SEZ (Fuentealba et al., 2015; Furutachi et al., 2015). The development of the hippocampal dentate gyrus is longer than in other brain areas. Progenitor cells from the embryonic dentate neuroepithelium migrate out of this zone through the dentate migratory stream and occupy several transient germinal niches before finally settling in a newly formed abventricular SGZ, transforming into quiescent SGZ NSCs mainly postnatally (Seki et al., 2014; Nicola et al., 2015; Matsue et al., 2018; Berg et al., 2019; Morales and Mira, 2019; Nelson et al., 2020). Adult NSCs share many features with embryonic aRGs, including a polarized morphology and the expression of common markers such as nestin, brain lipid-binding protein (BLBP), glutamate/aspartate transporter (GLAST) and the transcription factor Sox2 (Lagace et al., 2007; Ninkovic et al., 2007; Suh et al., 2007; Giachino et al., 2014), and are often referred to as radial glia-like NSCs. At the transcriptome level, single-cell RNA sequencing (scRNA-seq) and conventional RNA-seq studies show that adult NSCs are also closely related to, but distinct from, mature astrocytes (Beckervordersandforth et al., 2010; Llorens-Bobadilla et al., 2015; Hochgerner et al., 2018).

### Division Throughout Development

Cell division in neural progenitors and stem cells in the central nervous system has been elucidated using a combination of techniques. Key examples are selective lineage tracing; clonal analysis at single-cell resolution; and *in vivo* or whole-mount time-lapse imaging of *Drosophila* neuroblasts (Nbs), embryonic mammalian aRGs, and adult RG-like NSCs (Bossing et al., 1996; Schmidt et al., 1997; Urbach and Technau, 2004; Gao et al., 2014; Taverna et al., 2014; Doe, 2017; Cardenas et al., 2018; Cardenas and Borrell, 2019). Early during gestation, NECs first divide symmetrically and later asymmetrically to produce neuroblasts in the fly and aRGs in the mammalian brain (Figure 1; Gotz and Huttner, 2005; Kriegstein and Alvarez-Buylla, 2009; Brand and Livesey, 2011). In turn, aRGs initially divide symmetrically in the ventricular zone, generating more aRGs. They then switch to producing neurons either through direct neurogenesis, in which the aRG divides asymmetrically to self-renew and generate a neuron, or through indirect neurogenesis to generate various intermediate neural progenitors (INPs) with proliferative capacity, which amplifies neuronal production (Taverna et al., 2014; Cardenas and Borrell, 2019).

The orientation of the cleavage plane determines symmetric vs. asymmetric division (Gotz and Huttner, 2005) and is also important in the proper seeding of future adult NSCs during development (Falk et al., 2017). The indirect mode of asymmetric neurogenesis leads to the formation of an embryonic subventricular zone, where these INPs migrate before the neurons are ultimately produced (Haubensak et al., 2004; Miyata et al., 2004; Noctor et al., 2004). Indirect neurogenesis predominates in humans and other primates with expanded cortices, where additional types of progenitors are formed (Cardenas and Borrell, 2019). In the mouse, this mode is predominant in the neocortex but limited in the olfactory bulb (Cardenas et al., 2018; Cardenas and Borrell, 2019).

**FIGURE 2:**
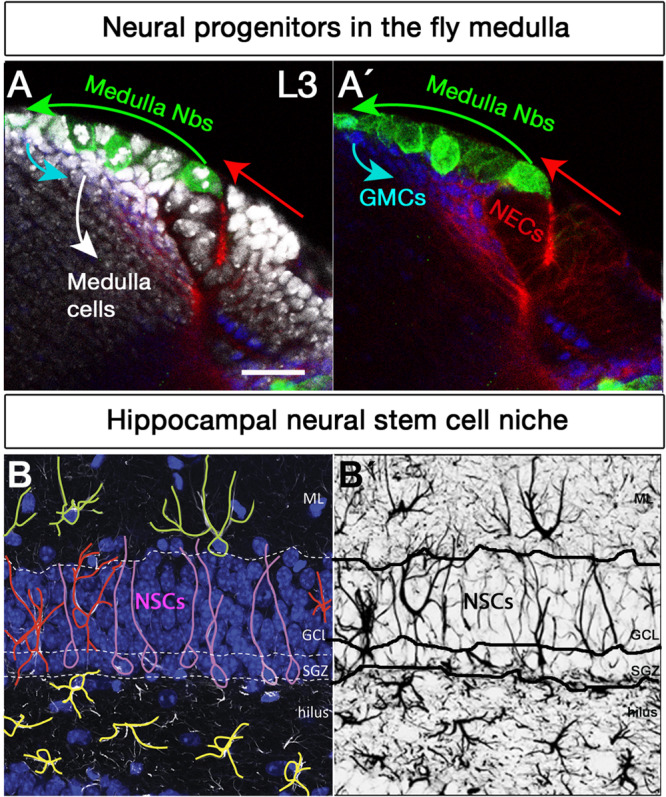
Neural stem cell niches in the *Drosophila* larval medulla and adult mouse hippocampus. **(A,A’)** Neural stem cell niche in the larval medulla: **(A)** neuroepithelial cells (NECs, *D*E-Cadherin, red) in the outer proliferation center, and their transition into medulla neuroblasts (Nbs, Miranda, green), ganglion mother cells (GMCs, Prospero, blue) and medulla postmitotic cells (DAPI, gray). Arrows indicate transition direction. Scale bar = 20 μm. **(B,B’)** Neural stem cell niche in the adult mouse hippocampus: **(B)** Neural stem cells with radial glia-like morphology (pink) are located in the hippocampal dentate gyrus (GFAP, white; DAPI, blue). Their soma sits at the border of the densely packed granule cell layer (GCL), the so-called subgranular zone (SGZ). Their primary process extends across the GCL and reaches the inner molecular layer (ML). NSCs express the markers Sox2, Prominin 1, Nestin (not shown) and glial fibrillary acidic protein GFAP (white), among others, and are mostly quiescent. Surrounding the NSCs a variety of highly branched niche astrocytes located in different layers are found. Those in the ML, GCL, and hilus are shown in green, red, and yellow, respectively. Mature astrocytes do not proliferate and express markers such as glial glutamate transporter 1 (GLT1), S100β (not shown) and GFAP (white), among others. Other niche elements such as blood vessels, INPs and neurons are not shown. **(B’)** GFAP immunostaining, marking both NSCs and astrocytes with distinctive morphologies.

Similarly, *Drosophila* neuroblasts undergo distinct types of cell division to shape different areas of the fly brain (Figures 1C,D). Type I neuroblasts are the most abundant neuroblast in the embryonic central brain (CB) and ventral nerve cord, and in the CB and optic lobes (Figures 2A,A’) of larval *Drosophila*. Type II neuroblasts exist in sets of eight in each brain lobe. Type I and II neuroblasts have been broadly studied (Doe, 2017). Both types divide asymmetrically; the main difference between them is that Type I neuroblasts produce ganglion mother cells (GMCs) directly, whereas neurogenesis from Type II neuroblasts is mediated by INPs, which then produce GMCs, which ultimately divide symmetrically to generate two neurons or glia (Bello et al., 2008; Boone and Doe, 2008; Bowman et al., 2008). In the embryonic ventral nerve cord, the equivalent of the vertebrate spinal cord, most neuroblasts begin in Type I mode and then they switch to Type 0 mode, where each Type 0 neuroblast divides asymmetrically multiple times and produces progeny that differentiate directly into neurons (Baumgardt et al., 2014). Conversely, in the tip of the outer proliferation center (t-OPC), larval neuroblasts transit from Type 0 to Type I mode to generate diverse cell types in the adult optic lobe (Bertet et al., 2014). A Type III neuroblast has recently been described in the larval optic lobe (Mora et al., 2018). These distal inner proliferation center (d-IPC)-derived neuroblasts show the particularity that, like the SEZ NSCs (Obernier et al., 2018), they undergo symmetric self-renewal to produce two identical progenies that retain neuroblast markers and produce T4 and T5 lobula plate neurons. The identification of these Type III neuroblasts has generated some controversy, and their existence has not been corroborated in other studies (Apitz and Salecker, 2018; Pinto-Teixeira et al., 2018). Future research will be necessary to confirm the presence of this Type III novel neuroblast division mode.

### Division in Adult Neurogenic Niches

Intermediate neural progenitor-mediated amplification of neuronal production also characterizes adult neurogenic niches (Figure 1B). Adult NSCs in the SEZ were long thought to behave like developmental aRGs, predominantly adopting an asymmetric neurogenic division mode as the main strategy to produce differentiated progeny while maintaining a pool of stem cells, before becoming depleted through a terminal symmetric division (Calzolari et al., 2015). However, this view has been recently challenged. Instead, it has been proposed that adult SEZ NSCs engage in two types of coexisting divisions, 20% of them undergoing symmetric self-renewal to contribute to the stem cell reservoir and the other 80% undergoing symmetric consuming divisions that produce transient amplifying progenitors, a type of intermediate neural progenitor (INP/TAP) (Obernier et al., 2018), which, in turn, generate a large variety of olfactory bulb interneurons (Merkle et al., 2007, 2014). This division mode allows the uncoupling of self-renewal and differentiation. The transition between the two NSC pools remains unclear.

In the adult SGZ niche (Figures 2B,B’), most divisions of radial glia-like NSCs are asymmetric (Figure 1B), giving rise to NSCs and dividing progenitors that will later become neurons (Encinas et al., 2011). However, symmetric self-renewing divisions have also been detected by *in vivo* clonal analysis with genetic marking (Bonaguidi et al., 2011). Recent live-imaging data suggests that radial glia-like NSCs follow a temporal developmental-like program upon activation, comprising an initial proliferative (symmetric) phase followed by a neurogenic (asymmetric) phase (Pilz et al., 2018). Active radial glia-like NSCs likely retain a molecular memory of their history and return to a less dormant quiescent state (Urban et al., 2016; Blomfield et al., 2019; Urban et al., 2019).

Adult NSCs in the SGZ will give rise to only one type of excitatory neuron (the dentate gyrus granule neuron) and, to a lesser extent, will produce local astroglial cells (Suh et al., 2007; Bonaguidi et al., 2011). After undergoing a series of neurogenic asymmetric divisions, radial glia-like NSCs become exhausted and terminally differentiate into mature astrocytes. This gliogenic process is poorly defined but is exacerbated during aging (Encinas et al., 2011; Gebara et al., 2016; Diaz-Moreno et al., 2018; Martin-Suarez et al., 2019) and in pathology (Sierra et al., 2015). Intriguingly, the terminal conversion of radial glia-like NSCs into astrocytes has not yet been captured by live imaging (Pilz et al., 2018), so additional studies are required to elucidate this pathway.

In summary, the presence of INPs is conserved in the fly and in mammalian adult neurogenic niches as a strategy to produce lineages with more neurons, in ways that resemble indirect neurogenesis in the developing mammalian brain. During adulthood, the dynamics of NSCs in the SGZ recapitulate the irreversible switch from a symmetrical self-renewing phase to an asymmetrical neurogenic division phase that characterizes cortical development. Conversely, in the SEZ, two subtypes of NSCs seem to coexist based on their symmetric mode of division. The molecular basis of these division modes, and the number of times adult NSCs divide before depletion, remains elusive.

## Intrinsic Control of Neural Progenitor Fate: Temporal and Regional Patterns

### Production of Cell Types in *Drosophila*

Neural stem cells can proliferate and differentiate into various cell types in response to both intrinsic factors and extrinsic cues from their stem cell niche. Spatial patterning plays a key role in acquiring NSCs identities in the developing nervous system. In the early embryo, the combined action of segment polarity, dorso-ventral, columnar and Hox genes act in gradients along the AP and DV axes and form an orthogonal grid that regionally divides the ventral nerve cord neuroectoderm and specifies the neuroblast identity (Table 1; Skeath and Thor, 2003; Technau et al., 2006; Urbach and Technau, 2008; Estacio-Gomez and Diaz-Benjumea, 2014). Interestingly, neuronal subtype specification in the vertebrate hindbrain and spinal cord relies on Hox-dependent regionalization of progenitor and postmitotic cells along the rostrocaudal axis as well in response to opposing morphogen gradients (Sagner and Briscoe, 2019), indicating that spatial colinearity is conserved among vertebrates and *Drosophila* (Philippidou and Dasen, 2013). In the larval brains, optic lobe neurons originate from two neuroepithelia, called the outer (OPC, Figures 2A,A’) and inner proliferation centers (IPC) (Hofbauer and Campos-Ortega, 1990). The OPC that gives rise to neuroblasts (Egger et al., 2007) is patterned into spatial domains along the anterior–posterior axis by expression of the transcription factors Visual system homeobox 1 (Vsx1), Optix and retinal homeobox (Rx), and the signaling molecules decapentaplegic (dpp), wingless (wg), and hedgehog (hh) (Table 1; Erclik et al., 2008, 2017; Gold and Brand, 2014).

**TABLE 1:** Intrinsic factors and glial-derived extrinsic signals influencing cell decisions in *Drosophila* and mammalian neurogenic niches.

Names/symbols	Human orthologs	Gene groups and pathways	Description
**In *Drosophila*:**			
***Intrinsic factors***			
Abdominal A (abd-A)	HOXA6 HOXC6	Bithorax complex HOX-like homeobox TFs	Required for segmental identity of the second through eighth abdominal segments
Abdominal B (Abd-B)	HOXA11 HOXD11	Bithorax complex HOX-like homeobox TFs	Specifies the identity of the posterior abdominal segments
Antennapedia (Antp)	HOXB7	Antennapedia complex HOX-like homeobox TFs	Regulates segmental identity in the mesothorax
Asense (ase)	ASCL1 ACSL2	Basic helix-loop-helix TFs	tTF in d-IPC Type I Nbs
Atonal (ato)	ATOH7	Basic helix-loop-helix TFs	tTF in d-IPC Type I and III Nbs
Baboon (babo)	TGFβR1	TGF-β type I receptors	Required for proliferation of Nbs
Broad (br)	BTBD18	C2H2 zinc finger TFs	tTF in thoracic later-born neurons
Castor (cas)	CASZ1	C2H2 zinc finger TFs	tTF in VNC Type I Nbs, thoracic Type I Nbs, CB Type II Nbs and INPs
Chronologically inappropriate morphogenesis (Chinmo)	BTBD18	C2H2 zinc finger TFs	tTF in thoracic early-born neurons
Dachshund (dac)	DACH1	Other DNA binding domain TFs	tTF in d-IPC Type I Nbs
Decapentaplegic (dpp)	BMP2	Bone morphogenetic proteins signaling pathway core components	Patterns the dorsal surface of the embryo and is expressed in a subset of Rx^+^ tOPC NECs
Deformed (Dfd)	HOXC4	Antennapedia complex HOX-like homeobox TFs	Involved in proper morphological identity of the maxillary segment and the posterior half of the mandibular segment
Dichaete (D)	SOX12 SOX14 SOX21	High mobility group box TFs	tTF in Me, tOPC and d-IPC Type I Nbs, CB Type II Nbs and INPs
Distal-less (Dll)	DLX1 DLX6	NK-like homeobox TFs	Expressed in Wg^+^ tOPC NECs and tTF in tOPC Type 0 Nbs
Dorsal (dl)	RELA RELB	Nuclear factor-κB	Patterns the ventral side of the embryo
Drop (Dr)	MSX2	NK-like homeobox TFs	Specifies the dorsal portion of the neuroectoderm
Engrailed (en)	EN1	NK-like homeobox TFs	Segment polarity gene involved in compartment identity and boundary formation
Epidermal growth factor receptor (EGFR)	EGFR	Receptor tyrosine kinases	Required for expansion of OPC NECs and patterns the ventral side of the embryo
Eyeless (ey)	PAX6	Paired homeobox TFs	tTF in Me and tOPC Type I Nbs, CB Type II Nbs and INPs
Grainy head (grh)	GRHL1	Polycomb group recruiters/DNA-binding proteins	tTF in CB Type II Nbs and INPs
Gooseberry (gsb)	PAX3	Paired homeobox TFs	Expressed in segmentally repeating pattern to define the A/P polarity of embryonic segments
Hedgehog (hh)	SHH DHH	Hedgehog signaling pathway core component	Marks ventral half of the OPC NECs
Homothorax (hth)	MEIS1 MEIS2	Tale homeobox TFs	tTF in Me Type I Nbs
Hunchback (hb)	IKZF5	C2H2 zinc finger TFs	tTF in VNC Type I Nbs
IGF-II mRNA-binding protein (Imp)	IGF2BP1 IGF2BP2 IGF2BP3	mRNA-binding protein	tTF in thoracic early-born neurons
Intermediate neuroblasts defective (ind)	GSX1	HOX-like homeobox TFs	Specifies the intermediate portion of the neuroectoderm
Klumpfuss (Klu)	ZBTB7A	C2H2 zinc finger TFs	tTF in Me Type I Nbs
Kruppel (Kr)	BCL6	C2H2 zinc finger TFs	tTF in VNC Type I Nbs
Labial (lab)	HOXA1 HOXB1	Antennapedia complex HOX-like homeobox TFs	Specifies derivatives of gnathocephalic segments
Optix	SIX3 SIX6	Six/Sine oculis homeobox TFs	Marks the adjacent ventral and dorsal main regions to Vsx1^+^ OPC NECs
Retinal Homeobox (Rx)	RAX	Paired-like homeobox TFs	Marks the tOPC NECs
POU domain protein 2 (Pdm2)	POU2F3	POU homeobox TFs	tTF in VNC Type I Nbs
Proboscipedia (pb)	HOXA2 HOXB2	Antennapedia complex HOX-like homeobox TFs	Required for the formation of labial and maxillary palps
Seven up (svp)	NR2F2	Nuclear receptor TFs	tTF in CB Type II Nbs and INPs
Sex combs reduced (Scr)	HOXA5	Antennapedia complex HOX-like homeobox TFs	Required for labial and first thoracic segment development
Sloppy paired 1 (slp 1)	FOXG1	Fork head box TFs	tTF in Me and tOPC Type I Nbs
Syncrip (Syp)	HNRNPR SYNCRIP	mRNA-binding protein	tTF in thoracic later-born neurons
Tailless (tll)	NR2E1	Nuclear receptor TFs	tTF in Me Type I Nbs
Ultrabithorax (Ubx)	HOXB6	Bithorax complex HOX-like homeobox TFs	Controls development of the posterior thoracic and first abdominal segments
Ventral nervous system defective (vnd)	NKX2-2	NK-like homeobox TFs	Specifies the ventral portion of the neuroectoderm
Visual system homeobox 1 (Vsx1)	VSX2	Paired-like homeobox TFs	Expressed in central OPC NECs
Wingless (wg)	WNT1	Wnt-TCF signaling pathway core component	Segment polarity gene involved in controlling the segmentation pattern of embryos by affecting the posterior cells of each parasegment and is expressed in a second subset of Rx^+^ tOPC NECs
*Niche/glia-derived factors*			
Activin-β (Actβ)	INHBA INHBB	TGFβ superfamily ligand	Secreted from surface glia
Anachronism (ana)	–	Glycoprotein	Secreted from cortex glia
Dally-like (dlp)	GPC4	Heparan sulfate proteoglycan Glypican (Membrane tethered)	Secreted from surface glia
Drosophila insulin-like peptides 1–8s (dILP1–8s)	IGF1/2	Insulin-like peptides	Secreted from cortex and surface glia
Glass bottom boat (gbb)	BMP7	Bone morphogenetic proteins signaling pathway ligand	Secreted from surface glia
Jelly belly (jeb)	–	Ligand of anaplastic lymphoma kinase	Secreted from glia
Spitz (Spi)	TGF-α	EGFR signaling pathway ligand	Secreted from cortex glia
Terribly reduced optic lobes (trol)	HSPG2	Heparan sulfate proteoglycan Perlecan (ECM component)	Secreted from surface glia

**Symbols/names**	***Drosophila* orthologs**	**Gene groups and pathways**	**Description**

**In mammals:**			
***Intrinsic factors***			
Castor zinc finger 1 (CasZ1)	Cas	C2H2 zinc finger TFs	tTF in the specification of late-born cell types in the retina
COUP-TF interacting protein 2/B cell leukemia/lymphoma 11B (Citp2/BCL11B)	CG9650	C2H2 zinc finger TFs	Specification of Layer V neurons
Distal-less homeobox 2 (Dlx2)	Dll	NK-like homeobox TFs	Regional specification (embryonic subpallium (LGE and MGE) and lateral postnatal/adult SEZ)
Empty spiracles homeobox 1 (Emx1)	ems	NK-like homeobox TFs	Regional specification (embryonic pallium and dorsal postnatal/adult SEZ)
Empty Spiracles Homeobox 2 (Emx2)	ems	NK-like homeobox TFs	Dentate gyrus regional identity
Eomesodermin (Tbr2)	Doc1	T-Box TFs	Specification of INPs
Eukaryotic translation initiation factor 4E nuclear import factor 1 (4E-T/EIF4ENIF1)	4E-T	eIF4E/mRNA translation regulator	Translational repression of neuronal specification TFs
Fez family zinc finger 2 (Fezf2)	erm	C2H2 zinc finger TFs	Specification of Layer V neurons
Forkhead box G1 (Foxg1)	Slp2	Fork head box TFs	Specification of deep-layer neurons
GS homeobox 2 (Gsh2/Gsx2)	ind	HOX-like homeobox TFs	Regional specification (embryonic subpallium (LGE and MGE) and dorsolateral postnatal/adult SEZ)
HOP homeobox (Hopx)	–	Homeobox TFs	Dentate gyrus regional identity
IKAROS family zinc finger 1 (Ikzf1)	Hb	C2H2 Zinc finger TFs	tTF in the specification of early-born cell types in the cortex and retina
Lysine (K)-specific methyltransferase 2A (Mll1/KMT2B)	trx	Trithorax complex	Preservation of regional identity
Lymphoid enhancer binding factor 1 (Lef1)	pan	High mobility group box TFs	Dentate gyrus regional identity
Neurogenic differentiation 1 (Neurod1)	amos ato	Proneural basic helix-loop-helix TFs	Required for neuronal differentiation
Neurogenin 2 (Neurog2)	tap	Proneural basic helix-loop-helix TFs	Drives differentiation of NSCs into neurons
NK2 homeobox 1 (Nkx2-1)	scro	NK-like homeobox TFs	Regional specification (embryonic subpallium (MGE), and ventrolateral and medial postnatal/adult SEZ)
Nuclear receptor subfamily 2, group F, member 1 (Nr2f1/COUP-TFI)	svp	Nuclear receptor TFs	Specification of upper-layer neurons
Paired box 6 (Pax6)	ey	Paired homeobox TFs	Expressed in Radial glia/NSCs; regional specification (embryonic pallium, dorsal postnatal/adult SEZ)
POU domain, class 3, transcription factor 3 (Brn1/POU3F3)	vvl	POU homeobox TFs	Specification of upper-layer neurons
Pumilio RNA-binding family member 2 (Pum2)	pum	RNA-binding family	Translational repression of neuronal specification TFs
Special AT-rich sequence binding protein 2 (Satb2)	dve	CUT homeobox TFs	Specification of upper-layer neurons
SRY (sex determining region Y)-box 5 (Sox5)	Sox102F	High mobility group box TFs	Specification of layer VI neurons
T-box brain transcription factor 1 (Tbr1)	Doc1	T-Box TFs	Specification of layer VI neurons
Transducin-like enhancer of split 4 (Tle4)	gro	Transcriptional corepressor	Specification of deep-layer neurons
Zinc finger E-box binding homeobox 2 (Sip1/Zeb2)	zfh1	C2H2 zinc finger TFs	Feedback signaling from neurons to progenitors
Zinc finger protein of the cerebellum 1 (Zic1)	opa	C2H2 zinc finger TFs	Regional specification (embryonic medial subpallium and septal postnatal/adult SEZ)
*Niche/Astroglia-derived factors*			
Insulin-like growth factor binding protein 6 (IGFBP6)	–	Regulation of insulin-like growth factor receptor signaling pathway	Secreted by non-neurogenic astroglia
Insulin-like growth factor 1 (Igf1)	dilp2	Insulin-like growth factor ligand	Systemic/niche factor
Interleukin 1 beta (IL-1β)	–	Cytokine activity	Secreted by SGZ niche astroglia
interleukin 6 (IL-6)	–	Cytokine activity	Secreted by SGZ niche astroglia
Jagged 1 (Jag1)	Ser	Notch signaling pathway membrane-bound ligand	Expressed by forebrain astroglia
Neurogenesin-1/Chordin-like protein 1 (Ng1/Chrdl1)	–	BMP antagonist	Secreted by SGZ niche astroglia
Secreted frizzled-related protein 4 (sFRP4)	–	Wnt antagonist	Secreted by OB astroglia
Thrombospondin 1 (Thbs1)	Tsp	Glycoprotein (ECM component)	Secreted by forebrain astroglia
Wingless-type MMTV integration site family (Wnt3,Wnt7a)	wg	Wnt pathway ligand	Secreted by SGZ/SEZ niche astroglia

*CB, central brain; d-IPC, distal inner proliferation center; ECM, extracellular matrix; LGE, lateral ganglionic eminence; Me, medulla; MGE, medial ganglionic eminence; Nbs, neuroblasts; NECs, neuroepithelial cells; NSCs, neural stem cells; OB, olfactory bulb; SEZ, subependymal zone; SGZ, subgranular zone; TFs, transcription factors; tTF, temporal transcription factor; t-OPC, tip of the outer proliferation center; VNC, ventral nerve cord.*

**FIGURE 3:**
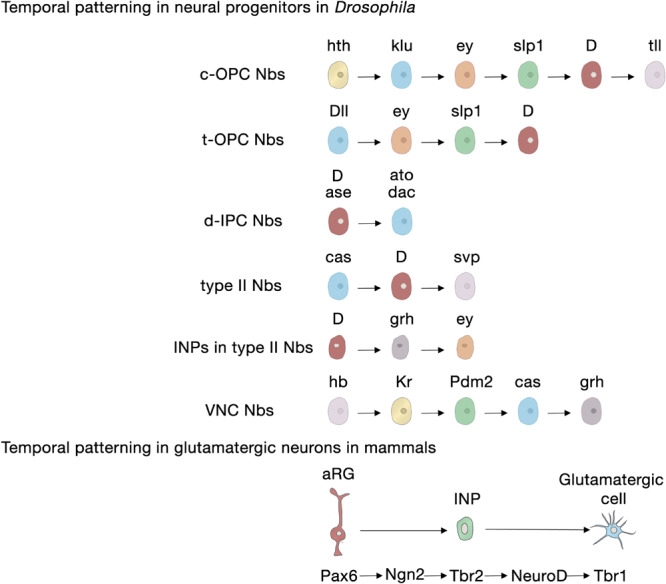
Temporal patterning in neural progenitors in *Drosophila* and in mammals. *Upper panel*, transcription factor sequences expressed in embryonic and larval *Drosophila* neural progenitors. *Lower panel*, core transcription factor sequence expressed in glutamatergic neurogenesis in the developing cerebral cortex, adult SGZ neurogenesis and adult OB glutamatergic juxtaglomerular interneuron neurogenesis. aRG, apical radial glia; c-OPC, central outer proliferation center; d-IPC, distal inner proliferation center; INP, intermediate neural progenitors; Nbs, neuroblasts; t-OPC, tip of the outer proliferation center; VNC, ventral nerve cord.

Neural progenitors and NSCs also generate distinct neuronal and glial subtypes over time (Figure 3). This generation of diversity in the developing brain depends on the sequential expression of transcription factors, a phenomenon known as temporal patterning (Doe, 2017; Holguera and Desplan, 2018) that was first observed in the embryonic *Drosophila* ventral nerve cord (Kambadur et al., 1998; Brody and Odenwald, 2000; Grosskortenhaus et al., 2005; Baumgardt et al., 2009; Doe, 2017). Indeed, temporal patterning is how the neural progenitors in *Drosophila* generate cellular diversity in different areas of the brain (Morante and Desplan, 2008; Erclik et al., 2017; Konstantinides et al., 2018). For example, Type I neuroblasts of the larval central outer proliferation center (c-OPC) express six different transcription factors as they age: homothorax (hth), klumpfuss (klu), eyeless (ey), sloppy paired 1 (slp1), Dichaete (D), and tailless (tll) (Table 1; Morante et al., 2011; Li et al., 2013; Suzuki et al., 2013). These temporal series are not unique and, for example, larval neuroblasts at the t-OPC express Distal-less (Dll), ey, Slp1, and D (Bertet et al., 2014) while neuroblasts from the d-IPC express asense (ase), D, atonal (ato), and dachshund (dac) (Table 1; Apitz and Salecker, 2018; Mora et al., 2018; Pinto-Teixeira et al., 2018).

Embryonic and larval Type II neuroblasts, and their derived INPs, have adopted a different strategy to increase neural diversity in the adult central complex. In their case, both neuroblasts and INPs express their respective sequences of temporal transcription factors that remain identical as they age from the embryonic to the larval stage. These are castor (cas), D, and seven up (svp) in neuroblasts; and D, grainyhead (grh) and ey in INPs (Table 1; Bayraktar and Doe, 2013; Walsh and Doe, 2017; Alvarez and Diaz-Benjumea, 2018).

However, not all temporal transcription factor sequences remain identical during the embryonic and larval stages. Type I neuroblasts in the embryonic ventral nerve cord and thoracic larval neuroblasts, which delaminate from the embryonic neuroectoderm of the ventral nerve cord, also sequentially express transcription factors, but these sequences differ between the animals’ embryonic and larval lives. Embryonic ventral nerve cord neuroblasts express a complex series of transcription factors [hunchback (hb), krueppel (Kr), POU domain protein 2 (Pdm2), Cas and grh], but thoracic Type I neuroblasts only express Cas and give rise to a series of early-born neurons expressing the BTB transcription factor Chinmo and the RNA-binding protein Imp, and later-born neurons expressing broad (Br) and another RNA-binding protein, Syp (Zhu et al., 2006; Maurange et al., 2008). Svp, an orphan nuclear hormone belonging to the COUP-TF family, triggers this temporal transition from Imp and Chinmo expression to Syp and Br expression by terminating Cas expression (Table 1; Maurange et al., 2008; Ren et al., 2017; Syed et al., 2017; Kanai et al., 2018).

**FIGURE 4:**
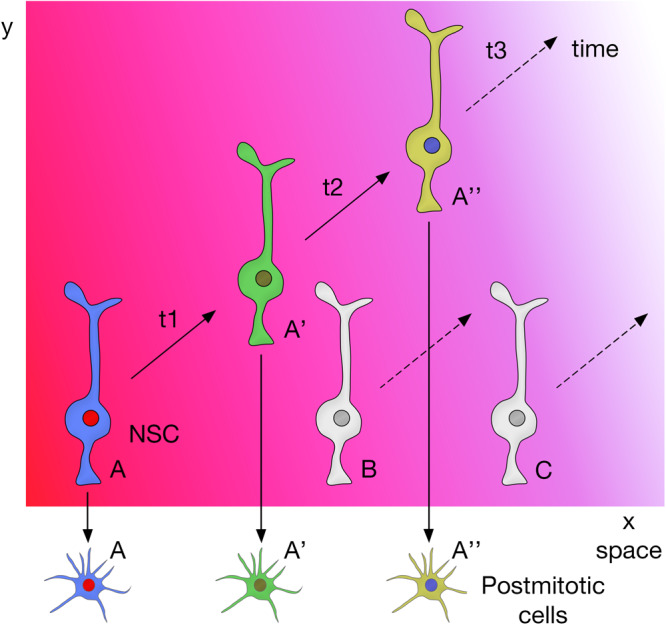
Spatial and temporal patterning on NSCs act together to promote neural diversity in the central nervous system.

In summary, these studies show that combinatorial inputs from the temporal and spatial axes act together to promote neural diversity in the central nervous system (Figure 4).

### Production of Neurons During Development in Mammals

In contrast to *Drosophila* neural progenitors, the temporal sequence of transcription factors in mammalian NSCs during development is less well understood. In the following section we will focus on the current understanding of cortical glutamatergic projection neuron production, a highly stereotyped process in which early-generated neurons occupy the deep layers of the cortex (V and VI) and late born neurons occupy the upper layers (II–IV) (Angevine and Sidman, 1961; Rakic, 1972). In this lineage, the transcription factor Pax6 specifically expressed in radial glia participates in the patterning of the telencephalon and in instructing neurogenesis through a cascade of transcription factors (Pax6 → Ngn2 → Tbr2 → Tbr1) that are sequentially expressed in radial glia cells, INPs and postmitotic glutamatergic projection neurons (Figure 3; Gotz et al., 1998; Stoykova et al., 2000; Heins et al., 2002; Englund et al., 2005). Tbr2, the transcriptional target of Ngn2, exerts key functions in this cascade: it directly represses Pax6 to favor the transition between cellular stages, from radial glia to INP; it promotes differentiation through the activation of projection neuron transcription factors including Tbr1, Ctip2, and Satb2 (Elsen et al., 2013; Kovach et al., 2013; Mihalas et al., 2016) and it regulates regional identity through the repression of ventral genes (Kovach et al., 2013). In addition, from the well-established gene repression network involved in the specification of deep neurons (Sox5, Tbr1, Fezf2, Ctip2) and upper-layer neurons (Satb2) within the mammalian cortex (Table 1; Britanova et al., 2008; Kwan et al., 2008; McKenna et al., 2011; Srinivasan et al., 2012; Leone et al., 2015), only Fezf2 has been detected in cortical progenitors (Hirata et al., 2004) and the rest are expressed in postmitotic neurons. Upper-layer competence has been linked to deep-layer neurogenesis. It is determined primarily through the repression of Tbr1 and derepression of Fezf2 by Foxg1 to acquire deep-layer identity, and the posterior feedback signal from deep-layer neurons to repress Fezf2/Ctip2 (Toma et al., 2014). Brn1 and Brn2 are also involved in this transition, as upper-layer neurons fail to be generated in Brn1/2 double mutants (Dominguez et al., 2013). Besides transcriptional regulation, another additional regulatory layer to ensure appropriate upper- versus deep-layer cortical neuron identities (Brn1 and Tle4) involves regulation at the translational level by the Pum2/4E-T repressor complex from aRGs that are transcriptionally primed to generate diverse types of neurons (Zahr et al., 2018).

Despite the lack of an unequivocal temporal transcription factor sequence in cortical neural progenitors, the COUP-TF family is still required for the temporal specification of mammalian neural progenitors. Knockdown of COUP-TFI/II in the mouse neocortex causes sustained neurogenesis and prolonged generation of early-born neurons, preventing the onset of gliogenesis (Naka et al., 2008). Other mammalian temporal factors include Ikaros, the ortholog of Hb, which specifies early-born neural identity in the cortex and retina (Elliott et al., 2008), and CasZ1, the ortholog of Cas, which specifies late-born neurons in the mammalian retina (Mattar et al., 2015). These studies reveal an overall conserved strategy regulating temporal identity transitions from flies to mammals and highlight the existence of very precise modes of gene expression control.

### Production of New Neurons During Adulthood

The generation of neuronal diversity relies largely on the regional patterning experienced by the cells during development, which, in turn, depends on morphogen gradients. In mice, SEZ NSCs are found in the lateral, medial and dorsal walls of the lateral ventricles, in the rostral migratory stream that directs the new neurons to the olfactory bulb, and in the olfactory bulb core itself (Figure 1B; Merkle et al., 2007; Alonso et al., 2008; Moreno-Estelles et al., 2012; Mizrak et al., 2019). NSCs in these different locations produce a variety of interneuron subtypes in the olfactory bulb due to a mosaic of patterned progenitor domains: ventral NSCs are the source of deep granule neurons and calbindin-positive periglomerular neurons, whereas NSCs in dorsal domains generate superficial granule neurons and dopaminergic periglomerular neurons (Merkle et al., 2007). Ultimately, this complex organization depends on the positional information integrated by the NSCs during development that correlates with the expression of a transcription factor code (Merkle et al., 2007; Fuentealba et al., 2015). For instance, pallial markers such as Emx1 and Pax6 are expressed in the dorsal domains; subpallial markers such as Dlx2, Gsh2, and Nkx2-1 are expressed in the lateral and ventral domains, respectively; and septal ventricular markers like Zic1/2 are expressed in the medial domains (Table 1; Kohwi et al., 2007; Young et al., 2007; Winpenny et al., 2011; Azim et al., 2012; Lopez-Juarez et al., 2013; Merkle et al., 2014; Delgado and Lim, 2015; Fiorelli et al., 2015; Tiveron et al., 2017). Interestingly, a population of glutamatergic juxtaglomerular excitatory OB interneurons that end up in the external plexiform layer are produced in the SEZ through the conserved Pax6 → Ngn2 → Tbr2 → NeuroD → Tbr1 transcription factor sequence (Brill et al., 2009; Roybon et al., 2009a; Figure 3), highlighting the conservation of this cascade for the specification of glutamatergic cell fate.

Cell-intrinsic programs are maintained even when ventral SEZ progenitors are heterotopically grafted into the dorsal SEZ or when they are cultured *in vitro* (Merkle et al., 2007), although cells can still switch fate when a single dorsal or lateral SEZ-enriched transcription factor is overexpressed (Azim et al., 2015). This indicates that the positional identity acquired by SEZ NSCs during development becomes independent of morphogen signaling in the adult brain. Recent data show that the preservation of this positional identity during adulthood involves a cell-autonomous epigenetic memory mechanism that depends on the chromatin regulator mixed-lineage leukemia 1 (Mll1) (Delgado et al., 2020), the mammalian homolog of the *Drosophila* gene trithorax (trx). Trx proteins are a heterogeneous group with varied activities mainly related to chromatin modification and remodeling to maintain active states and, thus, counteract the silencing activity of the polycomb group proteins (Piunti and Shilatifard, 2016; Schuettengruber et al., 2017). Classical genetics approaches in *Drosophila* revealed that both groups of proteins preserve the expression of Hox genes that determine anterior–posterior identities, although they are not involved in their induction (Geisler and Paro, 2015). In ventral SEZ NSCs, Trx/Mll1 is similarly required for maintaining Nkx2-1 expression, yet it does not participate in the initial induction of this transcription factor, which ultimately depends on the ventral morphogen sonic hedgehog (Shh) (Delgado et al., 2020).

Intriguingly, embryonic progenitors and adult NSCs located at equivalent sites and patterned similarly produce different progenies; for instance, aRGs in the dorsal pallium generate excitatory cortical neurons, whereas adult dorsal SEZ NSCs, related to these aRGs, produce inhibitory olfactory bulb interneurons (Fuentealba et al., 2015). Furthermore, there is a temporal pattern in the production of different subtypes of olfactory bulb interneurons, suggesting that different NSC domains dominate neuronal production at specific time points (Batista-Brito et al., 2008). The molecular program underlying these temporal switches has not been completely defined yet and future lineage tracing and scRNA-seq studies are required to solve the intricate codes that define SEZ NSC heterogeneity in time and space.

For producing new granule neurons in the adult hippocampal SGZ, the patterning information would also be acquired early during embryogenesis and preserved across development and into adulthood (Morales and Mira, 2019). SGZ NSCs are a continuum derived from progenitor cells that migrate out of the dentate neuroepithelium expressing the homeodomain-only protein, Hopx (Li et al., 2015; Berg et al., 2019) and the transcription factors Pax6 and Sox9 (Nelson et al., 2020). Recent data suggest that an early cohort of Tbr2 INPs expressing Notch ligands pioneers the subsequent NSC migration toward the newly formed outer (abventricular) SGZ niche, keeping neighboring NSCs in an undifferentiated state through Notch signaling (Nelson et al., 2020). The regional identity of SGZ NSCs is markedly influenced by a number of other transcription factors (Hatami et al., 2018), including Emx2 and Lef1 (Pellegrini et al., 1996; Galceran et al., 2000; Oldekamp et al., 2004), as well as by Wnt and bone morphogenetic protein signaling from the adjacent cortical hem (Li and Pleasure, 2007). Interestingly, the transcription factor sequence Pax6 → Ngn2 → Tbr2 → NeuroD → Tbr1 observed in developmental glutamatergic neurogenesis in the cortex is conserved along the lineage progression of adult SGZ neurogenesis (Figure 3; Hodge et al., 2008; Roybon et al., 2009b). Tbr2 likely facilitates the progression from the NSC to the INP state by directly binding and repressing Sox2 (Hodge et al., 2012).

Hopx-positive dentate progenitors upregulate cell membrane genes over development, pointing to a transition from an intrinsic mode of regulation in embryonic radial glia to an extrinsic, niche-dependent mode in adult RG-like NSCs (Berg et al., 2019). Similarly, sc-RNAseq data of adult hippocampal quiescent NSCs confirm that adult NSCs are enriched in genes encoding membrane-related proteins, pointing to an enhanced niche signaling integration capacity (Shin et al., 2015; Artegiani et al., 2017; Hochgerner et al., 2018). Furthermore, at least for some signaling pathways such as Notch, there is a switch in the expression of receptor subtypes in NSCs during the transition from development to adulthood that could influence the outcome of the signaling (Nelson et al., 2020).

## Extrinsic Control of Neural Progenitors

Neural progenitors and NSCs are also influenced by the local microenvironment where they reside, which determines their fate and self-renewal capacity (Morrison and Spradling, 2008; Siegenthaler et al., 2009; Lehtinen and Walsh, 2011; Lehtinen et al., 2011; Siegenthaler and Pleasure, 2011). The microenvironments in different brain regions and stages of development can be quite diverse, and this can be exploited as a strategy to generate cellular diversity.

Recent studies in the developing mouse forebrain have shown that transmission of temporal birthmarks from mother apical progenitors to their daughter cells fades with differentiation as environmental factors predominate (Vitali et al., 2018; Telley et al., 2019). A good example of this process occurs in developing layer IV neurons, whose final molecular identity and function is instructed by thalamocortical inputs (Pouchelon et al., 2014). Another remarkable illustration of microenvironmental influences is the production of signaling factors in postmitotic neurons. For example, neurotrophin-3 is regulated by the transcription repressor Sip1 (Zeb2), which feeds back to progenitors to modulate the timing of two cell fate switches during corticogenesis: neurogenesis to gliogenesis, and deep- to upper-layer neuron transitions (Seuntjens et al., 2009; Parthasarathy et al., 2014).

Classical transplantation experiments established that local environmental cues change over time and can control the competence of embryonic mammalian neural progenitors to produce neurons of different layers (McConnell, 1991). However, very few of these niches and their molecular signals have been characterized. An example of one that has is the extrinsic signaling from non-neural tissues, which has been proposed to co-ordinate neural progenitor and NSC proliferation in the developing mammalian forebrain (Siegenthaler and Pleasure, 2010; Lehtinen and Walsh, 2011; Lehtinen et al., 2011). In particular, retinoic acid signaling from the meninges was established to be important for switching from symmetric to asymmetric neurogenic proliferation in Foxc1-knockout mice (Siegenthaler et al., 2009). Additionally, meningeal cells organize the pial basement membrane, an extracellular matrix enriched in a variety of growth factors that covers the brain and might be involved in signaling at the basal side (Siegenthaler and Pleasure, 2011). Meanwhile, the apical side of embryonic and adult neural progenitors are in contact with the cerebrospinal fluid and the vascular system, and therefore might be influenced by extrinsic cues released from these non-neural tissues to regulate their self-renewal, differentiation and migratory capacity (Sawamoto et al., 2006; Tavazoie et al., 2008; Lehtinen and Walsh, 2011; Gato et al., 2019; Fame and Lehtinen, 2020). Indeed, isolated mouse apical progenitors cultured *in vitro* show only limited progression of temporal gene expression (Okamoto et al., 2016), suggesting that temporal progression in mammalian cortical progenitors may also require cell-extrinsic cues. This does not, however, seem to be the case with embryonic fruit fly neuroblasts. Isolated embryonic neuroblasts cultured *in vitro* express the same temporal sequences as observed *in vivo* (Grosskortenhaus et al., 2005), suggesting that the timing of temporal identity transitions in embryonic neuroblasts is regulated by an intrinsic mechanism (Doe, 2017), probably due to the short duration of the embryonic stage (24 h) and rapid divisions of embryonic neuroblasts compared to mammals.

### Adult Neurogenic Niches in Rodents

Heterotopic transplantation experiments demonstrated that cell-intrinsic programs tightly regulate SEZ NSCs (Merkle et al., 2007). Yet, despite all the intrinsic determinants, SGZ NSCs are highly plastic and their fate can be redirected when exposed to an adequate milieu (Suhonen et al., 1996). For instance, when transplanted into the rostral migratory stream, their progeny migrates to the olfactory bulb and differentiates into dopaminergic neurons (a non-hippocampal subtype), but when grafted into a non-neurogenic area, such as the cerebellum, they do not generate neurons (Suhonen et al., 1996). Similarly, grafting clonally expanded non-neurogenic NSCs from the spinal cord to the dentate gyrus results in the cells differentiating into new neurons that resemble resident hippocampal granule neurons, whereas cells grafted into non-neurogenic areas of the hippocampus either remain undifferentiated or give rise primarily to NG2 oligodendrocyte precursors, but not to neurons (Shihabuddin et al., 2000). This indicates that the grafted cells are instructed by local signals emanating from the neurogenic niche. A variety of factors released form the niche vasculature, choroid plexus, cerebrospinal fluid, ependymal cells, and local interneurons influence adult NSCs. Their role falls beyond the scope of the next section, that will instead focus on glial-derived niche signals.

## The Prevalent Role of Glial-Derived Signals

### Glial-Derived Signaling in *Drosophila*

**FIGURE 5:**
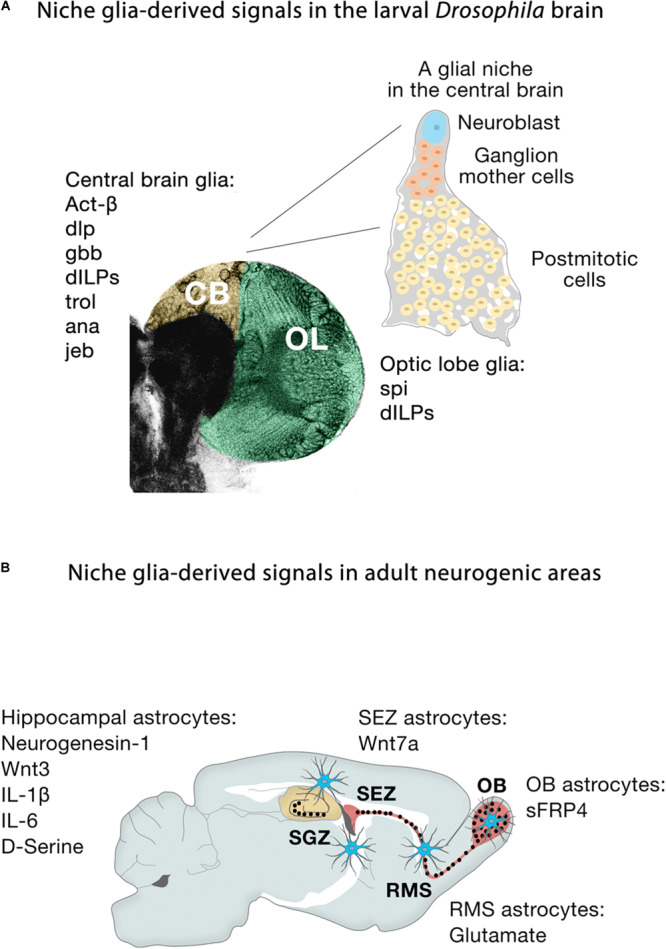
Niche glia-derived signals in the larval *Drosophila* brain and adult neurogenic areas. **(A)** Schematic representation of niche glia-derived signals in the larval central brain (CB, yellow) and optic lobe (OL, green), and schematic illustration of a GFP-labeled cortex glia Flp-out clone (gray) showing how the cortex glia ensheaths a Type I Nb (blue), ganglion mother cells (orange) and its neural progeny (yellow) in the central brain. **(B)** Schematic representation of niche astroglia-derived signals in the adult neurogenic areas. Astrocytes (blue cells) are regionally specified and secrete a variety of local signals to regulate neurogenesis. Astrocytes from the SEZ (the site of neuronal birth) secrete Wnt7a while olfactory bulb astrocytes (OB, the site of neuronal integration) express the Wnt antagonist sFRP4. Hippocampal astrocytes specifically release Wnt3, IL-1β, IL-6, and neurogenesin-1. Rostral migratory stream (RMS) astrocytes that ensheath migratory neuroblasts en route to the OB and hippocampal astrocytes also modulate adult neurogenesis through the supply of neurotransmitters (glutamate, D-serine).

Extrinsic signals from glia play important roles in microenvironments where they can act directly on different biological processes (Figure 5A). In larval *Drosophila* brains, cortex glia are the source of Spitz, a homolog of transforming growth factor-alpha, which is required for the initial proliferation of NECs in the medulla through the activation of the EGFR (Morante et al., 2013). Other glial-derived signals that regulate neuroblast proliferation in the developing larval brain include Activin-β (Act-β), via its receptor baboon (babo) (Zhu et al., 2008), dally-like (dlp), a heparan sulfate proteoglycan, and glass-bottom boat (gbb), a BMP homolog (Kanai et al., 2018). Surface and cortex glia also provide *Drosophila* insulin-like peptides (dILPs) in response to systemic nutritional cues (Chell and Brand, 2010; Sousa-Nunes et al., 2011; Lanet et al., 2013; Otsuki and Brand, 2017, 2018), and components of the extracellular matrix, such as trol, a secreted heparan sulfate proteoglycan Perlecan (Voigt et al., 2002; Park et al., 2003) required for timely reactivation of quiescent larval neuroblasts in the ventral nerve cord and CB. Conversely, secretion of the anachronism (ana) glycoprotein also affects the initiation of neuroblast proliferation, but in the opposite way: in *ana* mutants, mitotically regulated neuroblasts begin cell division too early (Ebens et al., 1993). Glial cells are not only necessary to regulate the proliferation of neuroblasts, but also protect the proliferation of neuroblasts under conditions of hypoxia and oxidative stress (Bailey et al., 2015), or nutrient restriction, through positive regulation of the jelly belly (jeb) secretion to stimulate Anaplastic lymphoma kinase (Alk)-dependent PI3K signaling in neuroblasts and protects their proliferation (Cheng et al., 2011). Release of dILPs by wrapping glia also stimulates lamina precursor cells to differentiate into lamina neurons in the visual system (Fernandes et al., 2017). These examples show the important function of glial-derived signaling within the stem cell niche in flies. Recent studies have also highlighted that establishing the correct niche architecture is necessary for encasing neural progenitor cells and NSCs and allowing them to divide (Morante et al., 2013; Speder and Brand, 2018).

### Niche Astroglial-Derived Signaling and Cell Heterogeneity in Rodents

During embryonic development in mammals, neurogenesis precedes gliogenesis, so the new neurons are generated in environments devoid of mature astroglial cells. However, in the adult, astrocytes are distributed throughout the neurogenic niches (Figure 5B), where they play fundamental roles. Gene expression profiling shows that astrocytes are heterogeneous across, and even within, regions (Doyle et al., 2008; Morel et al., 2017, 2019; Boisvert et al., 2018; Batiuk et al., 2020; Bayraktar et al., 2020), in line with the remarkable morphological and functional heterogeneity of astroglia throughout the brain (Emsley and Macklis, 2006; Matyash and Kettenmann, 2010). Specifically, co-culture experiments demonstrate that niche astrocytes (but not those from non-neurogenic areas such as the adult spinal cord) are regulators of all the stages along the neurogenic cascade, supporting NSC self-renewal, proliferation and neuronal differentiation of precursor cells through the release of soluble and/or cell membrane-bound factors (Lim and Alvarez-Buylla, 1999; Song et al., 2002). For instance, the molecular signatures of astroglia from the SEZ (the site of neuronal birth) and the olfactory bulb (the site of neuronal differentiation and maturation) are remarkably different: Wnt7a secreted by SEZ astrocytes promotes symmetric NSC self-renewing divisions, whereas its antagonist sFRP4 expressed by olfactory bulb astrocytes presumably blocks the activation of local olfactory bulb NSCs (Moreno-Estelles et al., 2012). In the SGZ, Wnt3, IL-1β, and IL-6 and the BMP antagonist neurogenesin-1 released by hippocampal astrocytes enhance neuronal fate specification and differentiation (Ueki et al., 2003; Lie et al., 2005; Barkho et al., 2006; Figure 5B). Non-neurogenic astrocytes instead secrete IGFBP6 (Barkho et al., 2006), which negatively regulates insulin growth factor (IGF)-II, an important player (together with IGF-I) in adult hippocampal neurogenesis (Bracko et al., 2012; Nieto-Estevez et al., 2016).

Hippocampal astrocytes can also negatively affect neurogenesis possibly through the cell membrane-bound Notch ligand, Jagged1 as suggested from *in vitro* experiments performed with astrocytes and NSCs isolated from the postnatal forebrain (Wilhelmsson et al., 2012). Astrocytes modulate the late phases of adult neurogenesis through the supply of neurotransmitters such as glutamate and D-serine. Blocking their exocytotic vesicular release from astrocytes or knocking-out NMDA receptors in neuroblasts compromises neuroblast survival during migration from the SEZ toward the olfactory bulb (Platel et al., 2010) and reduces dendritic spine maturation and synaptic integration of adult-born hippocampal neurons (Sultan et al., 2015). The extracellular matrix protein trombospondin-1 secreted by astrocytes, involved in astrocyte-induced synaptogenesis, is probably an astrocyte-derived factor that affects several steps of the neurogenic process, although its expression is not restricted to the niches (Lu and Kipnis, 2010). Other astrocyte-related factors regulating neuronal function throughout the brain may also contribute to the adequate functionality of the adult-born neurons once these neurons become mature, fully integrated and indistinguishable from their embryonically born counterparts.

Astrocytes may be more diverse than anticipated, with differences not only between distant regions such as the SEZ niche and olfactory bulb (Moreno-Estelles et al., 2012), but also possibly even within regions (Figures 2B,B’), including the hippocampal niche (Beckervordersandforth et al., 2014; Schneider et al., 2019). The advent of transcriptomics is unveiling the molecular basis for the plurality of astrocytes (Matias et al., 2019). A large scRNA-seq study uncovered seven regionally restricted astrocyte subtypes in the brain that correspond to developmental boundaries (Zeisel et al., 2018), while a more recent scRNA-seq dataset identified five distinct astrocyte subtypes in the cortex and hippocampus that are distinguished on the basis of their gene expression signature and topographic distribution (Batiuk et al., 2020). Another recent single-nucleus RNA-seq study of the hippocampus confirmed the existence of a complex atlas of astroglial cells with a continuous range of profiles and revealed the existence of an additional astrocyte state associated to aging and Alzheimer’s (Habib et al., 2020). Other studies have also uncovered the existence of intra-cortical astroglial heterogeneity and highlight layer-specific interactions between neurons and astrocytes (Lanjakornsiripan et al., 2018; Morel et al., 2019; Bayraktar et al., 2020), so it is conceivable that this holds true for other zones. A putative enrichment of specific astrocytic subtypes in defined subdomains of adult neurogenic areas may have interesting implications for our understanding of the functional interactions taking place between astrocytes, NSCs, newly born neurons, and pre-existing neurons.

## Future Perspectives of NSC Research

Single-cell RNA sequencing technology is revolutionizing how cell types are identified in developing and adult brains, providing astonishing insight into cellular diversity in specific regions including the optic lobe (Konstantinides et al., 2018), antennal lobe (Li et al., 2017), ventral nerve cord (Allen et al., 2020), and CB (Croset et al., 2018). In organisms with relatively simple brains, such as flies, whose brains consist of approximately 150,000 cells, the whole adult brain (Davie et al., 2018) can be investigated using this technology. And in animals with larger brains, like mice, the hypothalamus (Campbell et al., 2017; Romanov et al., 2017), lateral geniculate nucleus (Kalish et al., 2018), midbrain (La Manno et al., 2016), somatosensory cortex and hippocampus (Zeisel et al., 2015), visual cortex and anterior lateral motor cortex (Tasic et al., 2016, 2018); have been examined, among other areas. Some studies employ transgenic line–based sampling strategies and retrograde labeling of projection neurons to further assess the correspondence between the scRNA-seq identified cell types and specific cellular functions, including differential electrophysiological properties and long-range projection specificity (Zeisel et al., 2015; Tasic et al., 2016, 2018; Economo et al., 2018). Furthermore, spatiotemporal gene expression analysis of scRNA-seq datasets is revealing in unprecedented detail the intricate developmental trajectories that brain cells undergo through differentiation from embryonic neural progenitors (Telley et al., 2016, 2019; Nowakowski et al., 2017).

In the adult mouse, scRNA-seq studies have improved our understanding of the cellular composition of neurogenic niches. They have identified cellular states along the neurogenic lineage of the SEZ (Llorens-Bobadilla et al., 2015; Luo et al., 2015; Dulken et al., 2017; Zywitza et al., 2018; Mizrak et al., 2019) and the SGZ of the hippocampal dentate gyrus (Shin et al., 2015; Artegiani et al., 2017; Hochgerner et al., 2018). In *Drosophila*, a pioneer single-cell transcriptomic study has established a molecular cell atlas of the first instar larval brain (Brunet Avalos et al., 2019), identifying neurons expressing distinct neurotransmitters, neuromodulators and neuropeptides; neural progenitor cells; glial cells of different types; undifferentiated neurons; and non-neural cells.

Future studies will complement current knowledge and allow us to establish a detailed catalog of brain cell types (Ecker et al., 2017; Regev et al., 2017), as well as to fully map the cellular, molecular and spatial organization of the complex niche networks that maintain and regulate the division capacity of neural progenitors and stem cells. New data are already starting to shed light on the intrinsic epigenetic mechanisms that preserve regional identities in NSCs as the brain increases its complexity from development to adulthood. Additional studies are needed to clarify if there are glial subtypes in the niches and, if so, to analyze their possible role in regulating the different stages of the neurogenic cascade. It will be equally interesting to explore whether adult NSCs contribute to the intra-regional heterogeneity of astroglia and to generating their own local glial niche. Finally, we need to better understand how extrinsic cues received by neural progenitors are effectively interpreted to produce the correct intrinsic responses, as little is known about the specifics of these interactions.

## Author Contributions

HM and JM conceived the structure and content, and wrote the manuscript. Both authors contributed to the article and approved the submitted version.

## Conflict of Interest

The authors declare that the research was conducted in the absence of any commercial or financial relationships that could be construed as a potential conflict of interest.

## Abbreviations

aRG: apical radial glia
CB: central brain
GMC: ganglion mother cell
INP: intermediate neural progenitor
IPC: inner proliferation center
Nb: neuroblast
NEC: neuroepithelial cell
NSC: neural stem cell
OPC: outer proliferation center
scRNA-seq: single-cell RNA sequencing
SEZ: subependymal zone
SGZ: subgranular zone.
