# Corrigendum: Neurogenesis From Embryo to Adult – Lessons From Flies and Mice

**DOI:** 10.3389/fcell.2020.00686

**Published:** 2020-08-13

**Authors:** Helena Mira, Javier Morante

**Affiliations:** ^1^Instituto de Biomedicina de Valencia, Consejo Superior de Investigaciones Científicas, Valencia, Spain; ^2^Instituto de Neurociencias, Consejo Superior de Investigaciones Científicas y Universidad Miguel Hernandez, Alicante, Spain

**Keywords:** neurogenesis, neural stem cells, neural progenitors, niche, glia, intrinsic factors, extrinsic factors, adult neurogenesis

In the original article, there were six minor mistakes in Table 1 as published.

The gene group for Eyeless (ey) was stated as Paired is homeobox TFs.

The gene group for Gooseberry (gsb) was stated as Paired is homeobox TFs.

The gene group for Homothorax (hth) was stated as Tale is homeobox TFs.

The gene group for Optix was stated as Six/Sine oculis is homeobox TFs.

The gene group for Retinal Homeobox (Rx) was stated as Paired-like is homeobox TFs.

The gene group for Visual system homeobox 1 (Vsx1) was stated as Paired-like is homeobox TFs.

The correct gene groups are as follows:

The correct gene group for Eyeless (ey) is Paired homeobox TFs.

The correct gene group for Gooseberry (gsb) is Paired homeobox TFs.

The correct gene group for Homothorax (hth) is Tale homeobox TFs.

The correct gene group for Optix is Six/Sine oculis homeobox TFs.

The correct gene group for Retinal Homeobox (Rx) is Paired-like homeobox TFs.

The correct gene group for Visual system homeobox 1 (Vsx1) is Paired-like homeobox TFs.

The corrected Table 1 appears below.

**Table 1:** Intrinsic factors and glial-derived extrinsic signals influencing cell decisions in *Drosophila* and mammalian neurogenic niches.

**Names/symbols**	**Human orthologs**	**Gene groups and pathways**	**Description**
**In** ***Drosophila*****:**			
***Intrinsic factors***			
Abdominal A (abd-A)	HOXA6	Bithorax complex	Required for segmental identity of the second through eighth abdominal segments
	HOXC6	HOX-like homeobox TFs	
Abdominal B (Abd-B)	HOXA11	Bithorax complex	Specifies the identity of the posterior abdominal segments
	HOXD11	HOX-like homeobox TFs	
Antennapedia (Antp)	HOXB7	Antennapedia complex	Regulates segmental identity in the mesothorax
		HOX-like homeobox TFs	
Asense (ase)	ASCL1	Basic helix-loop-helix TFs	tTF in d-IPC Type I Nbs
	ACSL2		
Atonal (ato)	ATOH7	Basic helix-loop-helix TFs	tTF in d-IPC Type I and III Nbs
Baboon (babo)	TGFβR1	TGF-β type I receptors	Required for proliferation of Nbs
Broad (br)	BTBD18	C2H2 zinc finger TFs	tTF in thoracic later-born neurons
Castor (cas)	CASZ1	C2H2 zinc finger TFs	tTF in VNC Type I Nbs, thoracic Type I Nbs, CB Type II Nbs and INPs
Chronologically inappropriate morphogenesis (Chinmo)	BTBD18	C2H2 zinc finger TFs	tTF in thoracic early-born neurons
Dachshund (dac)	DACH1	Other DNA binding domain TFs	tTF in d-IPC Type I Nbs
Decapentaplegic (dpp)	BMP2	Bone morphogenetic proteins signaling pathway core components	Patterns the dorsal surface of the embryo and is expressed in a subset of Rx^+^ tOPC NECs
Deformed (Dfd)	HOXC4	Antennapedia complex	Involved in proper morphological identity of the maxillary segment and the posterior half of the mandibular segment
		HOX-like homeobox TFs	
Dichaete (D)	SOX12	High mobility group box TFs	tTF in Me, tOPC and d-IPC Type I Nbs, CB Type II Nbs and INPs
	SOX14		
	SOX21		
Distal-less (Dll)	DLX1	NK-like homeobox TFs	Expressed in Wg^+^ tOPC NECs and tTF in tOPC Type 0 Nbs
	DLX6		
Dorsal (dl)	RELA	Nuclear factor-κB	Patterns the ventral side of the embryo
	RELB		
Drop (Dr)	MSX2	NK-like homeobox TFs	Specifies the dorsal portion of the neuroectoderm
Engrailed (en)	EN1	NK-like homeobox TFs	Segment polarity gene involved in compartment identity and boundary formation
Epidermal growth factor receptor (EGFR)	EGFR	Receptor tyrosine kinases	Required for expansion of OPC NECs and patterns the ventral side of the embryo
Eyeless (ey)	PAX6	Paired homeobox TFs	tTF in Me and tOPC Type I Nbs, CB Type II Nbs and INPs
Grainy head (grh)	GRHL1	Polycomb group recruiters/DNA-binding proteins	tTF in CB Type II Nbs and INPs
Gooseberry (gsb)	PAX3	Paired homeobox TFs	Expressed in segmentally repeating pattern to define the A/P polarity of embryonic segments
Hedgehog (hh)	SHH	Hedgehog signaling pathway core component	Marks ventral half of the OPC NECs
	DHH		
Homothorax (hth)	MEIS1	Tale homeobox TFs	tTF in Me Type I Nbs
	MEIS2		
Hunchback (hb)	IKZF5	C2H2 zinc finger TFs	tTF in VNC Type I Nbs
IGF-II mRNA-binding protein (Imp)	IGF2BP1	mRNA-binding protein	tTF in thoracic early-born neurons
	IGF2BP2		
	IGF2BP3		
Intermediate neuroblasts defective (ind)	GSX1	HOX-like homeobox TFs	Specifies the intermediate portion of the neuroectoderm
Klumpfuss (Klu)	ZBTB7A	C2H2 zinc finger TFs	tTF in Me Type I Nbs
Kruppel (Kr)	BCL6	C2H2 zinc finger TFs	tTF in VNC Type I Nbs
Labial (lab)	HOXA1	Antennapedia complex	Specifies derivatives of gnathocephalic segments
	HOXB1	HOX-like homeobox TFs	
Optix	SIX3	Six/Sine oculis homeobox TFs	Marks the adjacent ventral and dorsal main regions to Vsx1^+^ OPC NECs
	SIX6		
Retinal Homeobox (Rx)	RAX	Paired-like homeobox TFs	Marks the tOPC NECs
POU domain protein 2 (Pdm2)	POU2F3	POU homeobox TFs	tTF in VNC Type I Nbs
Proboscipedia (pb)	HOXA2	Antennapedia complex	Required for the formation of labial and maxillary palps
	HOXB2	HOX-like homeobox TFs	
Seven up (svp)	NR2F2	Nuclear receptor TFs	tTF in CB Type II Nbs and INPs
Sex combs reduced (Scr)	HOXA5	Antennapedia complex	Required for labial and first thoracic segment development
		HOX-like homeobox TFs	
Sloppy paired 1 (slp 1)	FOXG1	Fork head box TFs	tTF in Me and tOPC Type I Nbs
Syncrip (Syp)	HNRNPR	mRNA-binding protein	tTF in thoracic later-born neurons
	SYNCRIP		
Tailless (tll)	NR2E1	Nuclear receptor TFs	tTF in Me Type I Nbs
Ultrabithorax (Ubx)	HOXB6	Bithorax complex	Controls development of the posterior thoracic and first abdominal segments
		HOX-like homeobox TFs	
Ventral nervous system defective (vnd)	NKX2-2	NK-like homeobox TFs	Specifies the ventral portion of the neuroectoderm
Visual system homeobox 1 (Vsx1)	VSX2	Paired-like homeobox TFs	Expressed in central OPC NECs
Wingless (wg)	WNT1	Wnt-TCF signaling pathway core component	Segment polarity gene involved in controlling the segmentation pattern of embryos by affecting the posterior cells of each parasegment and is expressed in a second subset of Rx^+^ tOPC NECs
*Niche/glia-derived factors*	INHBA	TGFβ superfamily ligand	Secreted from surface glia
Activin-β (Actβ)	INHBB		
Anachronism (ana)	–	Glycoprotein	Secreted from cortex glia
Dally-like (dlp)	GPC4	Heparan sulfate proteoglycan Glypican (Membrane tethered)	Secreted from surface glia
Drosophila insulin-like peptides 1–8s (dILP1–8s)	IGF1/2	Insulin-like peptides	Secreted from cortex and surface glia
Glass bottom boat (gbb)	BMP7	Bone morphogenetic proteins signaling pathway ligand	Secreted from surface glia
Jelly belly (jeb)	–	Ligand of anaplastic lymphoma kinase	Secreted from glia
Spitz (Spi)	TGF-α	EGFR signaling pathway ligand	Secreted from cortex glia
Terribly reduced optic lobes (trol)	HSPG2	Heparan sulfate proteoglycan Perlecan (ECM component)	Secreted from surface glia
**Symbols/names**	***Drosophila*** **orthologs**	**Gene groups and pathways**	**Description**
**In mammals:**			
***Intrinsic factors***			
Castor zinc finger 1 (CasZ1)	Cas	C2H2 zinc finger TFs	tTF in the specification of late-born cell types in the retina
COUP-TF interacting protein 2/B cell leukemia/lymphoma 11B (Citp2/BCL11B)	CG9650	C2H2 zinc finger TFs	Specification of Layer V neurons
Distal-less homeobox 2 (Dlx2)	Dll	NK-like homeobox TFs	Regional specification (embryonic subpallium (LGE and MGE) and lateral postnatal/adult SEZ)
Empty spiracles homeobox 1 (Emx1)	ems	NK-like homeobox TFs	Regional specification (embryonic pallium and dorsal postnatal/adult SEZ)
Empty Spiracles Homeobox 2 (Emx2)	ems	NK-like homeobox TFs	Dentate gyrus regional identity
Eomesodermin (Tbr2)	Doc1	T-Box TFs	Specification of INPs
Eukaryotic translation initiation factor 4E nuclear import factor 1 (4E-T/EIF4ENIF1)	4E-T	eIF4E/mRNA translation regulator	Translational repression of neuronal specification TFs
Fez family zinc finger 2 (Fezf2)	erm	C2H2 zinc finger TFs	Specification of Layer V neurons
Forkhead box G1 (Foxg1)	Slp2	Fork head box TFs	Specification of deep-layer neurons
GS homeobox 2 (Gsh2/Gsx2)	ind	HOX-like homeobox TFs	Regional specification (embryonic subpallium (LGE and MGE) and dorsolateral postnatal/adult SEZ)
HOP homeobox (Hopx)	–	Homeobox TFs	Dentate gyrus regional identity
IKAROS family zinc finger 1 (Ikzf1)	Hb	C2H2 Zinc finger TFs	tTF in the specification of early-born cell types in the cortex and retina
Lysine (K)-specific methyltransferase 2A (Mll1/ KMT2B)	trx	Trithorax complex	Preservation of regional identity
Lymphoid enhancer binding factor 1 (Lef1)	pan	High mobility group box TFs	Dentate gyrus regional identity
Neurogenic differentiation 1 (Neurod1)	amos	Proneural basic helix-loop-helix TFs	Required for neuronal differentiation
	ato		
Neurogenin 2 (Neurog2)	tap	Proneural basic helix-loop-helix TFs	Drives differentiation of NSCs into neurons
NK2 homeobox 1 (Nkx2-1)	scro	NK-like homeobox TFs	Regional specification (embryonic subpallium (MGE), and ventrolateral and medial postnatal/adult SEZ)
Nuclear receptor subfamily 2, group F, member 1 (Nr2f1/COUP-TFI)	svp	Nuclear receptor TFs	Specification of upper-layer neurons
Paired box 6 (Pax6)	ey	Paired homeobox TFs	Expressed in Radial glia/NSCs; regional specification (embryonic pallium, dorsal postnatal/adult SEZ)
POU domain, class 3, transcription factor 3 (Brn1/POU3F3)	vvl	POU homeobox TFs	Specification of upper-layer neurons
Pumilio RNA-binding family member 2 (Pum2)	pum	RNA-binding family	Translational repression of neuronal specification TFs
Special AT-rich sequence binding protein 2 (Satb2)	dve	CUT homeobox TFs	Specification of upper-layer neurons
SRY (sex determining region Y)-box 5 (Sox5)	Sox102F	High mobility group box TFs	Specification of layer VI neurons
T-box brain transcription factor 1 (Tbr1)	Doc1	T-Box TFs	Specification of layer VI neurons
Transducin-like enhancer of split 4 (Tle4)	gro	Transcriptional corepressor	Specification of deep-layer neurons
Zinc finger E-box binding homeobox 2 (Sip1/Zeb2)	zfh1	C2H2 zinc finger TFs	Feedback signaling from neurons to progenitors
Zinc finger protein of the cerebellum 1 (Zic1)	opa	C2H2 zinc finger TFs	Regional specification (embryonic medial subpallium and septal postnatal/adult SEZ)
*Niche/Astroglia-derived factors*			
Insulin-like growth factor binding protein 6 (IGFBP6)	–	Regulation of insulin-like growth factor receptor signaling pathway	Secreted by non-neurogenic astroglia
Insulin-like growth factor 1 (Igf1)	dilp2	Insulin-like growth factor ligand	Systemic/niche factor
Interleukin 1 beta (IL-1β)	–	Cytokine activity	Secreted by SGZ niche astroglia
interleukin 6 (IL-6)	–	Cytokine activity	Secreted by SGZ niche astroglia
Jagged 1 (Jag1)	Ser	Notch signaling pathway membrane-bound ligand	Expressed by forebrain astroglia
Neurogenesin-1/Chordin-like protein 1 (Ng1/Chrdl1)	–	BMP antagonist	Secreted by SGZ niche astroglia
Secreted frizzled-related protein 4 (sFRP4)	–	Wnt antagonist	Secreted by OB astroglia
Thrombospondin 1 (Thbs1)	Tsp	Glycoprotein (ECM component)	Secreted by forebrain astroglia
Wingless-type MMTV integration site family (Wnt3,Wnt7a)	wg	Wnt pathway ligand	Secreted by SGZ/SEZ niche astroglia

*CB, central brain; d-IPC, distal inner proliferation center; ECM, extracellular matrix; LGE, lateral ganglionic eminence; Me, medulla; MGE, medial ganglionic eminence; Nbs, neuroblasts; NECs, neuroepithelial cells; NSCs, neural stem cells; OB, olfactory bulb; SEZ, subependymal zone; SGZ, subgranular zone; TFs, transcription factors; tTF, temporal transcription factor; t-OPC, tip of the outer proliferation center; VNC, ventral nerve cord*.

The authors apologize for this error and state that this does not change the scientific conclusions of the article in any way. The original article has been updated.

